# Personals

**Published:** 1913-03

**Authors:** 


### PERSONALS.

Drs. Nelson W. Wilson and Edgar R. McGuire of Buffalo have been appointed to investigate the sanity of Edward Grimmell, the trephined burglar.

Dr. A. Crandall Way of Perry is taking the Panama trip. He has recently been adopted into the Seneca Tribe with the name of Gan-aja-wah, Big Kettle of the Beaver Clan. Ha-on-yu, also a Seneca, alias A. L. B., presents his congratulations.

Dr. T. G. Hogan of Lockport has been appointed jail physician of Niagara County at a salary of $240 a year. He succeeds Dr. Harry L. Parker.

Dr. R. S. Loop of Elmira has just returned from a sixteen days' trip to Panama and Cuba.

Dr. Henry Flood of Elmira has just returned from a two weeks' visit at Miami, Florida.

Dr. A. W. Booth of Elmira has left for a four weeks' trip to Panama, Cuba and Central America.

Dr. C. T. Crance of N. Tonawanda has returned from a week's trip to New York.

On January 25, Dr. Roland Meisenbach of Buffalo held a clinic in Toronto, by invitation. His topic was "Abbott's New and Rapid Method of Correcting the Fixed Types of Lateral Curvature of the Spine."

The honorary degree of D. C. L. has been conferred by Durham University on Sir William Osler, M. D., LL.D., Regius Professor of Medicine at Oxford University.

Dr. W. W. Britt of N. Tonawanda addressed the Shakespeare Club of his city on Scientific Achievement, February 3.

---

An organization meeting of the Y. W. C. A. was held at the office of Dr. Harris of Tonawanda, February 5.

---

Dr. Edward W. Roos of Corning visited his former home in Buffalo early in February.

---

Dr. S. Kavinoky has returned to Buffalo after spending a year in Berlin.

---

Dr. Regina Flood Keyes of Buffalo has returned from a trip around the world.

---

D. H. Squire, D. D. S., Dean of the Dental Department, University of Buffalo, has been elected President of the Institute of Dental Paedagogics.

---

Dr. G. H. Hitzel of Buffalo has been re-elected President of the German-Amercian Alliance.

---

Dr. Wm. L. Allen of North Tonawanda has been critically ill.

---

Dr. H. H. Glosser returned to Buffalo from a trip to North Carolina, early in February.

---

Dr. Herman E. Hayd of Buffalo is taking a month's trip to the West Indies, Isthmus and South America.

---

Sir George Turner, M. D., of England, became a martyr to science and humanity, while serving in the Leprosy Hospital in Pretoria, and has retired to his home in Devonshire, awaiting the end of this infection. It is just as well to remember cases of this sort and the noble work of others, more fortunate, or not engaged in hazardous research, in discussing the "effete aristocracy of Europe."

---

Dr. Jacob Goldberg of Buffalo has been elected chairman of the Board of School Examiners.

---

Captain Roswell Park, M. D., of Buffalo, of the Medical Reserve Corps, U. S. A., has recently delivered a course of lectures at the Army Medical School at Washington.

---

Dr. Lucien Howe of Buffalo will return shortly from a month's trip to Panama.

Dr. Richard J. Pearson of Buffalo will return from a cruise to Porto Rico about the middle of March.

---

A. F. Isham, D. D. S., has been elected President of the Dental Alumni of the University of Buffalo.

---

Dr. J. F. MacPherson of Buffalo was operated on for appendicitis at the Johns Hopkins Hospital, February 12, by Dr. M. T. Phinney.

---

Dr. W. B. Cochrane of Rochester has moved from 597 East Avenue to 1293 Park Avenue.

---

Dr. Louis Hengerer of New York, formerly of Buffalo, visited in Buffalo last month.

---

Dr. and Mrs. Pemberton Lundy of N. Tonawanda were injured in an automobile collision with a street car February 4. We are glad to state that the automobile was the most seriously damaged, and that Dr. and Mrs. Lundy escaped with slight injuries.

---

Dr. George W. Gregg of Canandaigua recently visited his summer cottage on the lake and found that several guns and other property had been stolen.

---

A satchel of instruments, belonging to Dr. N. E. Kavinoky of Buffalo, was stolen from his auto at the corner of Main and Mohawk streets, February 16. (Note—We print these announcements, without request, because a similar item resulted in the restoration of property, some months ago and some months after the announcement was made.)

---

Dr. Burt C. Johnson of Buffalo sailed for Panama March 1.

---

Dr. Frank H. Ransom of Buffalo will move to 880 W. Ferry street, May 1.

---

Dr. Hyatt Regester of Buffalo has purchased the large, modern house at 615 Elmwood avenue, which he will use as his office and home.

---

Dr. G. H. Sherman of Detroit returned early in February, from a five weeks' trip to Germany and England.

---

Dr. W. S. Grimes of Detroit has moved his office to the new Broadway Market Building.

